# Opposite Effects of Neuroprotective Cannabinoids, Palmitoylethanolamide, and 2-Arachidonoylglycerol on Function and Morphology of Microglia

**DOI:** 10.3389/fnins.2019.01180

**Published:** 2019-11-07

**Authors:** Urszula Hohmann, Markus Pelzer, Joshua Kleine, Tim Hohmann, Chalid Ghadban, Faramarz Dehghani

**Affiliations:** Department of Anatomy and Cell Biology, Medical Faculty, Martin Luther University Halle-Wittenberg, Halle (Saale), Germany

**Keywords:** 2-arachidonoylglycerol, palmitoylethanolamide, peroxisome proliferator-activated receptor, neuroprotection, microglial cells

## Abstract

Various studies performed in cultured cells and in *in vivo* models of neuronal damage showed that cannabinoids exert a neuroprotective effect. The increase in cannabinoids and cannabinoid like substances after stroke has been postulated to limit the content of neuronal injury. As well-accepted, inflammation, and neuronal damage are coupled processes and microglial cells as the main intrinsic immunological effector within the brain play a central role in their regulation. Treatment with the endocannabinoid, 2-arachidonoylglycerol (2-AG) or the endocannabinoid-like substance, palmitoylethanolamide (PEA) affected microglial cells and led to a decrease in the number of damaged neurons after excitotoxical lesion in organotypic hippocampal slice cultures (OHSC). 2-AG activated abnormal cannabidiol (abn-CBD) receptor, PEA was shown to mediate neuroprotection via peroxisome proliferator-activated receptor (PPAR)α. Despite the known neuroprotective and anti-inflammatory properties, the potential synergistic effect, namely possible entourage effect after treatment with the combination of these two protective cannabinoids has not been examined yet. After excitotoxical lesion OHSC were treated with PEA, 2-AG or a combination of both and the number of damaged neurons was evaluated. To investigate the role of microglial cells in PEA and 2-AG mediated protection, primary microglial cell cultures were treated with lipopolysaccharide (LPS) and 2-AG, PEA or a combination of those. Thereafter, we measured NO production, ramification index, proliferation and PPARα distribution in microglial cells. While PEA or 2-AG alone were neuroprotective, their co-application vanished the protective effect. This behavior was independent of microglial cells. Furthermore, PEA and 2-AG had contrary effects on ramification index and on NO production. No significant changes were observed in the proliferation rate of microglial cells after treatment. The expression of PPARα was not changed upon stimulation with PEA or 2-AG, but the distribution was significantly altered. 2-AG and PEA mediated neuroprotection was abolished when co-applied. Both cannabinoids exert contrary effects on morphology and function of microglial cells. Co-application of both cannabinoids with different targets did not lead to a positive additive effect as expected, presumably due to the contrary polarization of microglial cells.

## Introduction

Traumatic brain injury affects a high number of young adults and their hospitalization is still a significant public challenge (Meaney et al., 2014). During neuronal damage a complex series of mechanisms becomes activated (Dirnagl et al., 2003; Kunz et al., 2010). Energy failure leads to calcium overload, depolarization of neurons and release of neurotransmitters with consecutive excitotoxicity, induced through overstimulation from excitatory receptors like NMDA receptor. This cascade is followed by activation of proteolytic enzymes and release of reactive oxygen species, damaging mitochondria, followed by apoptosis and neuronal death (Bruce et al., 1995; Kunz et al., 2010). Injured neuronal tissue releases pro-inflammatory cytokines leading to migration of inflammatory cells to the damaged site and inflammation (Jassam et al., 2017). Microglial cells play a crucial role during this process of secondary neuronal damage since they are the immunocompetent cells of the central nervous system (Kettenmann et al., 2011).

Cannabinoids positively affect cellular and molecular processes during ischemic, excitotoxical, or traumatic brain injury and were shown to be protective in different models mostly via microglial cells. All kind, plant-derived (isolated from *Cannabis sativa*), endo- (animal-derived) and synthetic cannabinoids were shown to affect neuronal damage (Kreutz et al., 2007, 2009; Mechoulam and Shohami, 2007; Koch et al., 2011a, b; Grabiec et al., 2012; Fernández-Ruiz et al., 2015; Guida et al., 2017; Belardo et al., 2019; Impellizzeri et al., 2019). Using the model of excitotoxically lesioned OHSC the number of damaged neurons was significantly reduced after treatment with endocannabinoids, like *N*-arachidonoyl dopamine, 2-arachidonoylglycerol (2-AG) or PEA associated with altered microglial cell number but not the phytocannabinoid, Δ-9-tetrahydrocannabinol (Kreutz et al., 2007; Koch et al., 2011a; Grabiec et al., 2012).

2-AG was shown to induce protection after neuronal lesion and to reduce the amount of tumor necrosis factor α released from LPS activated microglial cells (Facchinetti et al., 2003). Both, PEA and 2-AG are produced in the central nervous system and upregulated after neuronal damage (Kondo et al., 1998; Panikashvili et al., 2001; Franklin et al., 2003) and were shown to exhibit neuroprotection in several *in vivo* and *in vitro* models (Koch et al., 2011a; Beggiato et al., 2018; Herrera et al., 2018). Anti-inflammatory effects of PEA were associated with PPARα activation (LoVerme et al., 2005; Koch et al., 2011a; Citraro et al., 2013), and induction of PPARα expression was related in parallel to protective effects (Genovese et al., 2008; Koch et al., 2011a). A recent study demonstrated the presence of PPARα in different brain regions on neurons, astrocytes and microglial cells (Warden et al., 2016). Effects of 2-AG were abolished by O-1918 and cannabidiol (CBD), both antagonists of the abn-CBD sensitive receptor (abn-CBDR) (Kreutz et al., 2009). Evidence for functional abn-CBDR in the brain was pharmacologically found on microglial cells (Franklin and Stella, 2003; Walter et al., 2003; Kreutz et al., 2009). Consequently, 2-AG mediated protection depends on the presence of microglial cells as confirmed by microglial cells depletion (Kohl et al., 2003; Kreutz et al., 2009).

2-AG mediated protective properties were demonstrated in a variety of animal models of degenerative neurological disorders including multiple sclerosis, Parkinson’s disease, and Alzheimer’s disease (Scotter et al., 2010; Pertwee, 2014; Mounsey et al., 2015) and *in vitro* in astrocytes exposed to oxygen-glucose deprivation (Wang et al., 2015, 2018). 2-AG is the most abundant endocannabinoid in the brain and known to bind and activate CB_1_ and CB_2_ receptors. The treatment with exogenous 2-AG attenuated neuronal damage *in vivo* partly via CB_1_ and mimicked the effects reported after aplication of synthetic CB_2_ agonists. Some effects were absent in CB_1_^–/–^ mice (Mechoulam and Shohami, 2007; Magid et al., 2019). Furthermore, Carrier et al. (2004) observed that 2-AG affected microglial cells via CB_2_. However, in rat OHSC and after NMDA damage effects of 2-AG were not blocked by CB_1_ or CB_2_ antagonists but inhibited by abnormal cannabidiol sensitive receptor (abn-CBDR) antagonists. These results make an involvement of CB_1_ or CB_2_ in 2-AG mediated neuroprotection unlikely (Kreutz et al., 2009). Application of PEA improved neuronal survival *in vitro* in primary mouse cortical astrocyte-neuron co-cultures (Beggiato et al., 2018) and in cortical neurons after hypoxia (Portavella et al., 2018). PEA possessed further beneficial properties in animal models of degenerative neurological disorders including vascular dementia (Siracusa et al., 2017). PEA and anandamide, if administrated together reduced the pain response 100-fold more potently than both substances alone and induced stronger vascular effects (Calignano et al., 1998; Ho et al., 2008). Such a co-application of two active cannabinoids increased their efficacy via the so called entourage effect, which is an endogenous cannabinoid molecular regulation route (Ben-Shabat et al., 1998). Ben-Shabat et al. (1998) demonstrated for the first time, that two inactive compounds, namely 2-linoleoylglycerol and 2-palmitoylglycerol potentiate the binding of 2-AG to the CB_1_ and thereby its effects. Additionally, 2-linoleoylglycerol significantly inhibited the inactivation of 2-AG. Similarly, PEA was reported to prevent the inactivation of anandamide (Jonsson et al., 2001; Ho et al., 2008) indicating possible entourage effect. Microglial cells, the main immune cell of the central nervous system, has a ramified morphology and is stationary, surveying its surrounding if in an undamaged and non-inflammatory state (Smith et al., 2012). In pathologies, like neuropathic pain CB_2_ expression increased in parallel to appearance of activated microglial cells. CB_2_ ligands significantly alleviated the pain indicating that microglial cells are a main target of cannabinoids (Zhang et al., 2003; Luongo et al., 2010). During neuronal damage microglial cells become amoeboid, migrate to the lesion site, proliferate and can be affected by cannabinoids, as for example PEA potentiates microglial cell motility (Franklin et al., 2003; Vinet et al., 2012). Microglial cells mediate neuroprotection, but can also contribute to the damage, e.g., by upregulation of iNOS, an enzyme producing toxic amounts of NO from L-arginine (Garry et al., 2015). 2-AG was shown to induce the iNOS expression and NO production (Lipina and Hundal, 2017) and to stimulate NO release in invertebrate immune cells via CB_1_ (Stefano et al., 2000). Contrary, NO donors were found to be neuroprotective (Kakizawa et al., 2007). Little is known about the influence of endocannabinoids on arginase, which inhibits the production of NO as a competing regulatory enzyme in the arginase-NO-synthase regulatory system in microglial cells. In peripheral immune cells Δ-9-tetrahydrocannabinol and AM1241, an CB_2_ agonist induced arginase 1 expression (Hegde et al., 2010; Ma et al., 2015).

Earlier studies consistently reported about increased levels of endocannabinoids, such as PEA or 2-AG after neuronal injury. A question is raised why the secondary neuronal damage can’t be prevented despite the high presence of neuroprotective substances. Since both endocannabinoids 2-AG and PEA if applied exogenously were shown to be neuroprotective via abn-CBDR or PPARα respectively, we asked whether the neuroprotective potential of both is additive. To assess a potential entourage effect between 2-AG and PEA on neuroprotective properties OHSC were excitotoxically lesioned and treated with PEA, 2-AG or combination of both. As mentioned, PEA and 2-AG target microglial cells. Therefore, their effects on function and morphology of primary microglial cells were investigated in untreated or LPS stimulated cultures. Ramification index, NO production, proliferation index, and temporal PPARα distribution were determined overtime.

## Materials and Methods

All experiments involving animal material were performed in accordance with the directive 2010/63/EU of the European Parliament and the Council of the European Union (22.09.2010) and approved by local authorities of the State of Saxony-Anhalt (permission number: I11M18, date: 01.12.2012) protecting animals and regulating tissue collection used for scientific purposes.

### Materials

2-Arachidonylglycerol (2-AG, 10 nM, stock solved in DMSO; Tocris, Minneapolis, MN, United States, cat No. 1298), Clodronate (100 μg/ml, stock solved in Aqua; Bayer Vital GmbH GB; PZN: 04299668), Palmitoylethanolamide (PEA, 10 nM, stock solved in DMSO, Tocris, cat No. 0879), LPS (10 ng/ml, stock solved in Aqua; Sigma-Aldrich, cat No. L8274) and NMDA (50 μM, stock solved in Aqua bidest., Sigma-Aldrich, cat No. M3262) were used and applied to the culture medium according to treatment protocol.

### Cell Culture

Primary microglia astrocyte co-cultures were prepared from 1 day old Wistar rats and cultured, as described earlier (Kohl et al., 2003; Grabiec et al., 2012). Briefly brains were treated with 0.5 mg/mL DNAse (Worthington, Bedford, MA, United States) and 4 mg/mL trypsin (Merck Millipore, Billerica, MA, United States) solved in Hank’s balanced salts solution (Invitrogen, Carlsbad, CA, United States). Cells were cultured in DMEM (Invitrogen, cat No. 41965-062) with 10% FBS (Invitrogen, cat No. 10500-062) and 1 ml streptomycin/penicillin (Invitrogen). After 10 days microglial cells were isolated from astrocytic monolayer and seeded into well plates.

For immunocytochemical analysis 50,000 cells were placed on glass cover slips coated with poly-L-lysin and allowed to attach for 3 h. Cannabinoids were applied for 48 h to determine the microglial cells morphology. Bromodeoxyuridine (BrdU) (0.01 mM, Sigma-Aldrich) was added to the culture medium 16 h before the fixation to assess proliferation. Intracellular distribution of PPARα was analyzed 1, 6, and 24 h after treatment. The cells were fixed with 4% paraformaldehyde (Sigma-Aldrich, Munich, Germany) for 10 min and stored in 0.02M PBS at 4°C for further analysis.

For NO measurement supernatant of 50,000 cells treated for 72 h with cannabinoids was collected and stored at −80°C until further analysis.

### Organotypic Hippocampal Slice Cultures (OHSC)

Organotypic hippocampal slice cultures were prepared from 7 to 9 day old Wistar rats as reported earlier (Grabiec et al., 2012, 2017; Hagemann et al., 2013; Hohmann et al., 2017, 2019) and kept at 35°C in a fully humidified atmosphere with 5% (v/v) CO_2_. Culture medium was changed every other day. After 6 days *in vitro* experiments were started. All slices despite the control groups were treated with NMDA (50 μM) for 4 h. OHSC were treated with PEA (10 nM) or 2-AG (10 nM) or their combination for 72 h. The NMDA treated set of excitotoxically damaged OHSC was supplemented with 2-AG, PEA or combination of both or left untreated to asses an effect on neuronal damage in region of dentate gyrus.

**FIGURE 1:**
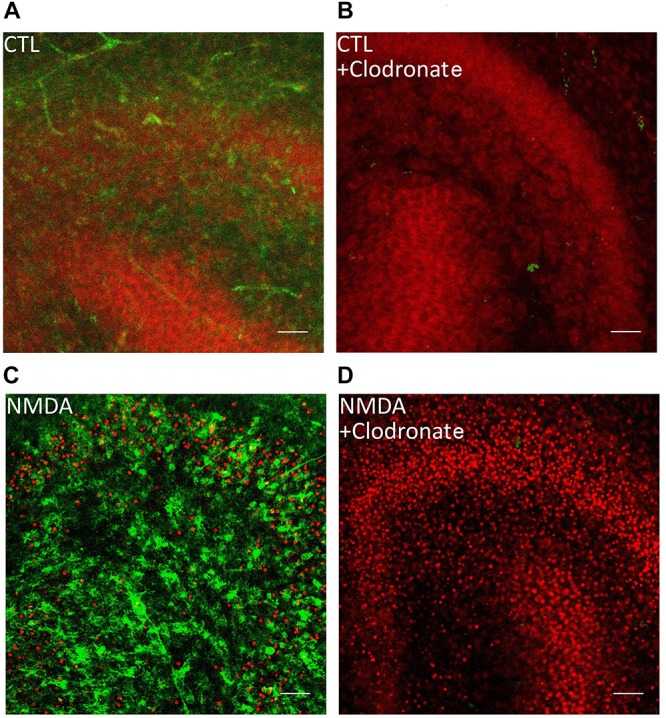
Clodronate depletes microglial cells from OHSC. **(A)** PI labeled control OHSC with dentate gyrus and CA3. No PI positive nuclei (red) and ramified IB_4_ (green) labeled microglial cells are visible. **(B)** Application of clodronate to CTL slices removed IB_4_ positive microglial cells without damaging the neurons. **(C)** Treatment with NMDA led to massive increase in PI positive cells and IB_4_ positive microglia. **(D)** Depletion of microglial cells led to exacerbation of neuronal damage after NMDA treatment in OHSC. Scale bar = 50 μm.

**FIGURE 2:**
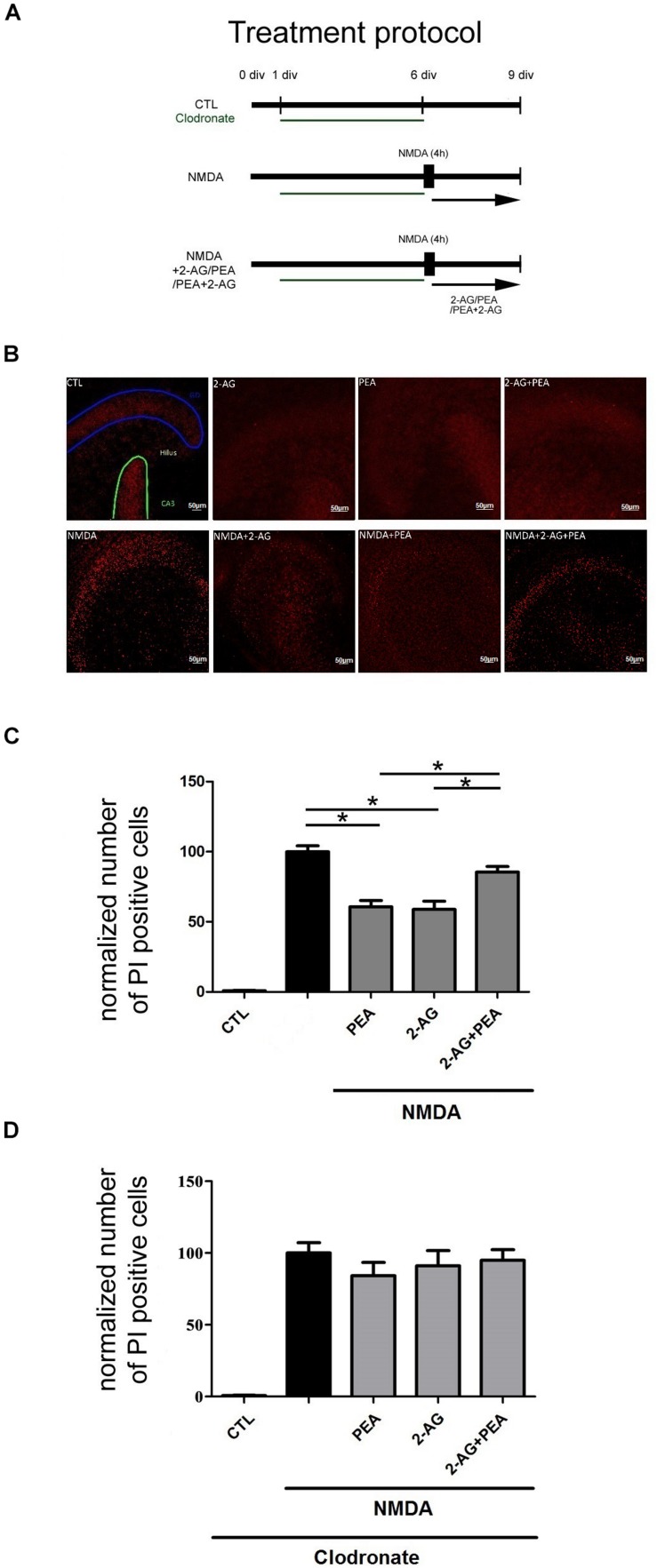
Effects of 2-AG and PEA in excitotoxically damaged OHSC. 2-AG and PEA did not further decrease the number of PI positive degenerated neurons in comparison to OHSC treated with 2-AG or PEA alone. **(A)** Treatment protocol. Control OHSC (CTL), were kept in culture medium and served as negative controls. The NMDA treated group function as positive control (NMDA). For microglial cells depleted groups clodronate (green) was applied from 1 div until 6 div. Cannabinoids were added alone on day 6 *in vitro* (div) to OHSC or following 4 h incubation with NMDA (NMDA+2-AG/PEA/PEA+2-AG). The fixation was performed on 9 div. **(B)** Representative images of the dentate gyrus in OHSC stained with PI (in red). After NMDA damage a massive increase in the number of damaged neurons occurred in comparison to control group (CTL), PEA, and 2-AG significantly reduced the neurodegeneration, but combination of both did not significantly reduce the number of PI positive nuclei. Dentate gyrus (GD) is highlighted in blue, Cornu ammonis (CA) 3 in green. Scale bar = 50 μm. **(C)** Quantitative analysis of PI positive nuclei in treated groups. The number of PI positive neurons increased significantly after treatment with NMDA (n_*NMDA*_ = 42) in comparison to control group (n_*CTL*_ = 42). 2-AG, PEA alone or combination of both had no effect on viability of OHSC (n_*PEA*_ = 12, n_2–AG_ = 12, n_2–AG__+__*PEA*_ = 10). Treatment with PEA (n_*NMDA*__+__*PEA*_ = 23) or 2-AG (n_*NMDA*__+__2–AG_ = 20) of excitotoxically damaged OHSC reduced significantly the number of PI positive cells. The combination of 2-AG and PEA after NMDA damage induced no significant protective effect (n_*NMDA*__+__2–AG__+__*PEA*_ = 34). **(D)** Treatment of microglia depleted OHSC with NMDA induced a massive neuronal damage (n_*CTL*_ = 15, n_*NMDA*_ = 18). The application of PEA, 2-AG or combination of both to clodronate treated NMDA damaged OHSC had no significant effect on the number of PI positive cells (n_*CLO*__+__*NMDA*__+__2–AG_ = 12, n_*CLO*__+__*NMDA*__+__*PEA*_ = 12, n_*CLO*__+__*NMDA*__+__2–AG__+__*PEA*_ = 14). Statistics was performed using a One-Way ANOVA with Bonferroni *post hoc* analysis and significance was chosen for *p* < 0.05. The asterisk denotes significant results regarding the respective measurement indicated with the bar. The values are served as a mean with standard error of the mean.)

To investigate the role of microglial cells OHSC were incubated with 100 μg/ml clodronate from 1 to 6 day *in vitro* (div). Clodronic acid, a bisphosphonate, affects only cells of the monocytic lineage and leads to apoptosis of microglia and macrophages (Kohl et al., 2003) (Figures 1, 2A).

All slice cultures were treated with propidium iodide (PI; Merck Millipore, cat No. 537059) 2 h prior to fixation to visualize degenerated neurons (Ebrahimi et al., 2010; Grabiec et al., 2012, 2017; Hezel et al., 2012).

### Immuno-, Lectinhistochemistry and Staining

All antibodies and lectins and conditions used are listed in Table 1. For labeling of incorporated BrdU fixed microglial cells were incubated with 2 mol/l HCl for 1 h, washed three times with PBS/Triton and pre-incubated with normal horse serum (Gibco BRL, Life Technologies, Eggenstein, Germany, cat No. 31874, dilution 1:20) in PBS/Triton. Afterward anti-BrdU antibody (Table 1) was applied for 1 h, followed by incubation with Alexa 488 conjugated goat anti-mouse antibody for 1 h. Cells were washed three times with PBS/Triton and incubated with 4′,6-diamidino-2-phenylindole (DAPI, Sigma-Aldrich, Munich, Germany, cat No. D9542).

**TABLE 1:** Antibodies.

**Name**	**Company**	**Number**	**Dilution**	**Antibody ID**
Biotinylated goat anti-rabbit antibody	Sigma-Aldrich	B7389	1:100	
BrdU	DAKO	M0744	1:100	
Alexa 488 conjugated IB_4_	Molecular Probes, Life Technologies, Eggenstein, Germany	I21411	1:500	AB_2314662
Biotin labeled IB_4_	Vector Laboratories, Burlingame, CA, United States	B-1205	1:100	AB_2314661
ExtrAvidin-Peroxidase	Sigma-Aldrich	E2886	1:100	
PPARα	Thermo Fisher, Waltham, MA, United States	PA1-822A	1:500	AB_2165595

Organotypic hippocampal slice cultures were stained with Alexa 488 conjugated IB_4_ (Molecular Probes). All fluorescence stained slides were washed with PBS/Triton and Aqua dest. before covering with mounting medium (DAKO, Agilent Technologies, Inc., Santa Clara, CA, United States).

For measurement of ramification index biotin labeled IB_4_ was used. To measure the PPARα distribution, an anti-PPARα antibody was applied as characterized before (Koch et al., 2011a). After washing with PBS, biotinylated goat anti-rabbit antibody was applied for 1 h. The following subsequent steps were the same as for IB_4_ staining: the cells were washed tree times with PBS and incubated with ExtrAvidin-Peroxidase for 1 h. After washing with PBS and Tris buffer, the slides were stained with 3,3′-Diaminobenzidine (DAB) (Sigma-Aldrich, cat No. D8001) and covered with Entallan (Merck Millipore, Darmstadt, Germany, cat No. 107960).

### Microscopy and Analysis

For analysis of proliferation index, ramification index and PPARα staining five areas per cover slip were recorded with Leica DMi8 (Wetzlar, Germany) or Axioplan (Zeiss, Oberkochen, Germany) microscopes.

Proliferation index was represented as the ratio of BrdU positive cells to all DAPI positive cells. The BrdU positive cells were counted using image J v1.46r (National Institutes of Health, Laboratory for Optical and Computational Instrumentation, University of Wisconsin, Madison, WI, United States).

To evaluate the ramification of microglial cells, the surface of microglia was stained with IB_4_ and the outlining of the cell was divided by the smallest convex hull around the cell. Values close to 1 correspond to strongly amoeboid cells while lower numbers represent ramified cells. The analysis was performed automatically using a self-written MatLab script (The MathWorks, Natick, MA, United States).

Next, the translocation of PPARα from the cytoplasm to the nucleus or vice versa was assessed after treatment with cannabinoids. PPARα staining was performed and manually evaluated by counting cells that showed a nuclear or/and cytoplasmic staining or were free of PPARα labeling. Results are presented as proportion of cells with a specific expression pattern relative to the total number of cells. For the calculation of the standard error of the mean in these experiments we used bootstrapping to calculate empirical standard deviations. Therefore all measurements were used and resampled 10,000 times. Afterward, the proportion of nuclear, cytoplasmic, nuclear+cytoplasmic location or no expression was calculated for each resampling and an empirical standard deviation and standard error of the mean was calculated.

The imaging of the fixed OHSC was performed using a confocal laser scanning microscope (LSM 510 Meta, Zeiss) with an excitation wavelength of 488 nm for IB_4_ and 543 nm for PI. Emission was detected using a band-pass filter with Δλ = 510–550 nm (IB_4_) and Δλ = 610–720 nm (PI). The dentate gyrus was visualized with a 20x objective, as a z-stack with a step width of 2 μm (Grabiec et al., 2012). The number of PI positive death cells in the obtained image stacks was analyzed using the maximal intensity projection and quantified using a self-written Matlab script.

### Nitrite Assay

A standard solution was prepared by solving sodium nitrite in medium up to concentrations of 100, 50, 25, 12.5, 6.25, 3.125, and 1.5625 μM. The measured values were used for calculation of a standard curve. 50 μl of the standard solutions or 50 μl of the collected samples were analyzed in duplicate. After applying 50 μl of Griess reagent (Sigma-Aldrich) the extinction was measured after 15 min at 540 nm in a microplate reader (SynergyTM Mx, BioTek Instruments, Winooski, VT, United States). The nitrite concentrations for the samples were interpolated from the standard curves.

### Statistical Analysis

Statistics was performed using the one-way ANOVA with Bonferroni post-test and significance was chosen for *p* < 0.05. All *p*-values refer to the respective controls of the same parameter of the same cell line or to the treatment with agonist for the respective receptor. All groups were normalized to the positive control. Statistics for cellular PPARα was performed using a Chi Square test and significance was chosen for *p* < 0.05. The values are served as a mean with standard error of the mean. The asterisk denotes significant results regarding the respective measurement indicated with the bar.

## Results

### 2-AG and PEA Do Not Further Decrease the Number of PI Positive Degenerated Neurons

The application of NMDA led to an increase in the number of PI positive cells in OHSC (100.0 ± 4.24%) in comparison to control group (0.86 ± 0.28%, Figures 2B,C).

Treatment of NMDA lesioned OHSC with PEA (60.64 ± 4.52%) or 2-AG (58.78 ± 5.94%) induced a significant reduction of neuronal damage. Combination of 2-AG and PEA (85.46 ± 3.92%) in excitotoxically damaged OHSC did not decrease the number of dead cells in a significant manner (Figures 2B,C). Notably the values were not significantly different when compared to NMDA group, but significantly higher when compared to NMDA+2-AG or NMDA+ PEA groups.

### Depletion of Microglial Cells Leads to Loss of Neuroprotection

The application of NMDA to microglia depleted OHSC (Figure 1) led to a significant increase in the number of PI positive cells (100.0 ± 7.12%) in comparison to control group (0.74 ± 0.17%, Figure 2D). The depletion of microglial cells was controlled through IB_4_ staining (Figure 1).

Neither the application of PEA (84.19 ± 9.29%) nor 2-AG (91.04 ± 10.68%) to NMDA damaged microglia depleted OHSC induced a significant neuroprotective effect. Also, the combination of 2-AG and PEA (94.93 ± 7.42%) to excitotoxically damaged microglia depleted OHSC showed any significant effect on the number of dead neurons (Figure 2D).

### Effects of 2-AG and PEA Treatment on Ramification Index

**FIGURE 3:**
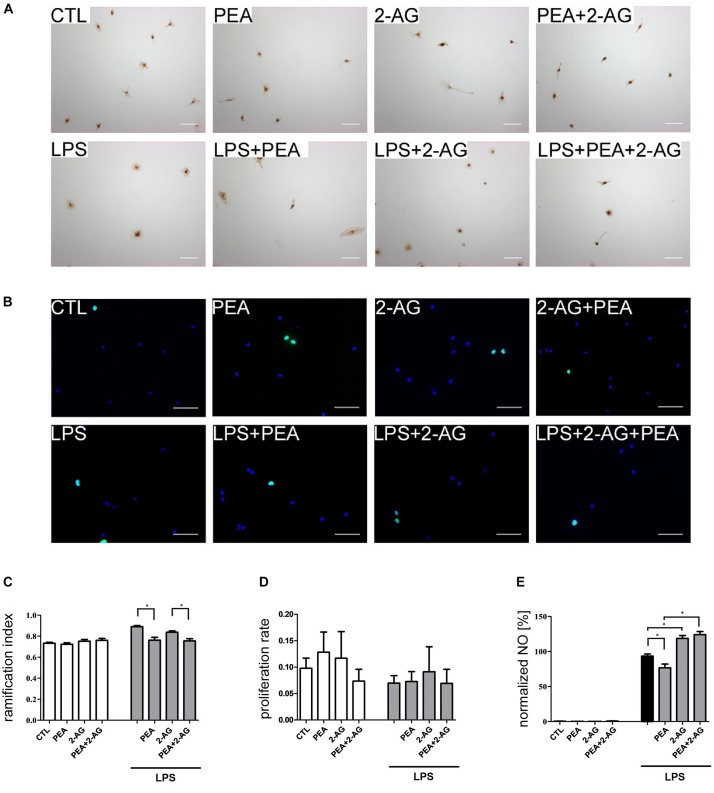
Effect of 2-AG and PEA on ramification index, proliferation index and nitrite concentration of primary rat microglia. Representative pictures of stained microglia for **(A)** ramification index (IB_4_) and **(B)** proliferation rate (BrdU in green, DAPI in blue). Scale bar = 50 μm. **(C)** Effects of 2-AG and PEA on ramification index. Application of LPS (n_*LPS*_ = 22) led to an increase in the ramification index in comparison to control group (CTL; n_*CTL*_ = 22). Whereas PEA co-applied with LPS (n_*LPS*__+__*PEA*_ = 23) reduced the ramification index significantly, 2-AG (n_*LPS*__+__2–AG_ = 23) had no effect. 2-AG, PEA, or combination of both applied alone (n_*PEA*_ = 19, n_2–AG_ = 22, n_2–AG__+__*PEA*_ = 22) had no effect on ramification index. The combination of LPS, 2-AG and PEA (n_*LPS*__+__2–AG__+__*PEA*_ = 22) led to decrease in the ramification index in comparison to the groups treated with LPS and 2-AG. **(D)** No effect of 2-AG and PEA with/or without LPS on proliferation of primary microglia cells could be observed (n_*CTL*_ = 12, n_*LPS*_ = 12, n_*PEA*_ = 11, n_2–AG_ = 11, n_2–AG__+__*PEA*_ = 12, n_*LPS*__+__*PEA*_ = 12, n_*LPS*__+__2–AG_ = 11, n_*LPS*__+__2–AG__+__*PEA*_ = 12). **(E)** Effects of 2-AG and PEA on nitrite concentration after 72 h. PEA, 2-AG, or PEA combined with 2-AG had no effect on the nitrite concentration in primary microglia (n_*CTL*_ = 18, n_*PEA*_ = 18, n_2–AG_ = 18, n_2–AG__+__*PEA*_ = 18). After administration of LPS, nitrite concentration increased significantly. PEA co-applied with LPS (n_*LPS*__+__*PEA*_ = 18) decreased the NO production in comparison to LPS (n_*LPS*_ = 18). 2-AG+LPS (n_*LPS*__+__2–AG_ = 18) and also 2-AG+PEA+LPS (_*nLPS*__+__2–AG__+__*PEA*_ = 18) treated microglia produced significantly more NO in comparison to LPS. Statistics was performed using a one-way ANOVA test with Bonferroni correction and significance was chosen for *p* < 0.05. The asterisk denotes significant results regarding the respective measurement indicated with the bar. The values are served as a mean with standard error of the mean.

Application of PEA (0.72 ± 0.01) or 2-AG (0.75 ± 0.02) had no impact on ramification index in comparison to control group (0.73 ± 0.01). Treatment with LPS (0.89 ± 0.01) led to a more amoeboid morphology of cells. Co-application of LPS and PEA reduced the ramification index significantly (0.76 ± 0.03) making cells more ramified. Incubation with 2-AG did not change the ramification index (0.84 ± 0.01) in comparison to LPS. However, PEA and 2-AG application together with LPS induced significant reduction in ramification index (0.76 ± 0.02) (Figures 3A,C).

### Effects of 2-AG and PEA on Nitrite Concentration

Application of PEA (0.19 ± 0.1%), 2-AG (0.14 ± 0.06%), or PEA+2-AG (0.76 ± 0.30%) did not change the nitrite concentration in comparison to control group (0.48 ± 0.22%). Treatment with LPS (100.0 ± 2.24%) led to an increased concentration of nitrite. Whereas co-application of LPS and PEA reduced the nitrite concentration significantly (81.06 ± 4.6%), incubation with 2-AG increased the nitrite concentration (128.2 ± 5.52%) in comparison to LPS. The same increase was detected for application of 2-AG+ PEA+ LPS (134.5 ± 7.11%) (Figure 3E).

### Effect of 2-AG and PEA on Proliferation of Primary Microglial Cells

After incubation with LPS (0.07 ± 0.01), PEA (0.13 ± 0.04), or 2-AG (0.12 ± 0.05) no significant changes in proliferation were detected in comparison to control group (0.097 ± 0.02). The results for PEA+2-AG (0.07 ± 0.02); LPS+PEA (0.07 ± 0.019); LPS+2-AG (0.09 ± 0.05); and LPS+PEA+2-AG (0.07 ± 0.03) were not significantly altered after treatment (Figures 3B,D). The overall test showed no significant differences between the groups.

### PPARα Distribution and Cellular Localization After Incubation With 2-AG and PEA

**FIGURE 4:**
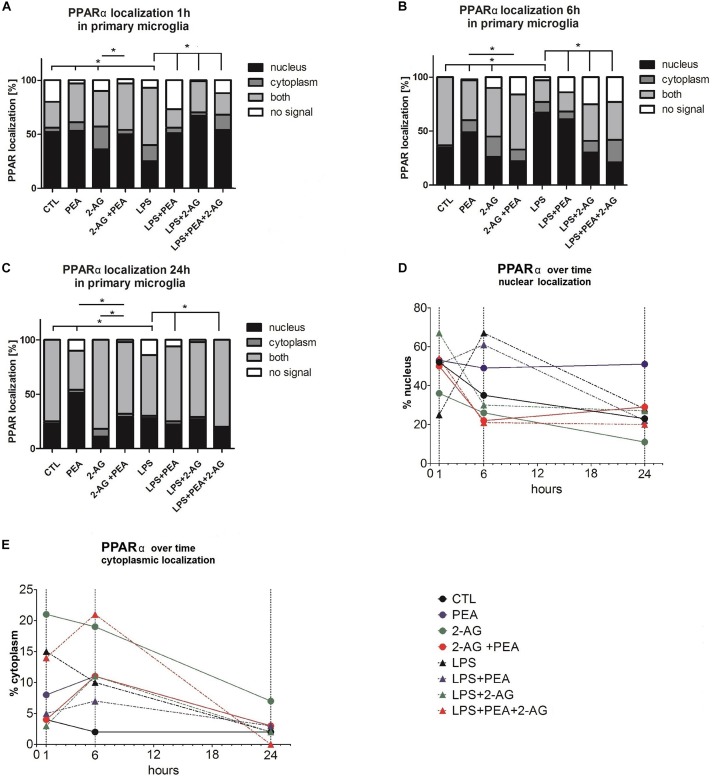
PPARα subcellular distribution and cellular localization after incubation with 2-AG and PEA in primary microglial cells. The localization of PPARα was analyzed over time by the use of staining **(A–C)** 1, 6, and 24 h after treatment, **(D)** nuclear, **(E)** cytoplasmic localization (1, 6, and 24 h after treatment). The number of cells which did not or expressed PPARα in the nucleus or/and cytoplasm was counted. In graphs **(A–E)** the subcellular location was scored in 483 (1 h), 362 (6 h), and 337 (24 h) cells by light microscopy and presented as percentage. Each independent experiment was repeated at least three times. The same data are displayed in two different ways to point out the differences in the distribution **(A–C)** between the time points for cytoplasmic and nuclear localization **(D,E)**. Statistics was performed using a Chi Square test and significance was chosen for *p* < 0.05. The asterisk denotes significant results regarding the respective measurement indicated with the bar. The values are served as a mean with standard error of the mean.

The evaluation of the localization of the PPARα receptor was significantly altered for treated groups overtime (1, 6, 24 h) (Figures 4A–E). The positive staining was localized in the cytoplasm and in the nucleus. The subcellular location was scored in 483 (1 h), 362 (6 h), and 337 (24 h) cells by light microscopy and presented as percentage. Each independent experiment was repeated at least three times (*n* = 3). The number of cells expressing nuclear as compared to cytoplasmic PPARα was significantly different between groups. Treatment with PEA; 2-AG; and LPS induced a significant shift in PPARα distribution after 1 and 6 h from nuclear to cytoplasmic and from cytoplasmic to nuclear localization. PEA and LPS changed significantly the localization of the receptor after 24 h (Figures 4A–C). The distribution was significantly different between 2-AG (Nucleus, N: 36%, Cytoplasm, C:21%, Both, B:33%, No signal, None:10%) and 2-AG co-applied with PEA (N:50%, C:4%, B:43%, None:4%) after 1 h, between PEA (N:49%, C:11%, B:38%, None:2%) and 2-AG+PEA (N:22%, C:11%, B:51%, None:16%) after 6 h and between PEA (N:51%, C:3%, B:36%, None:10%) or 2-AG (N:11%, C:7%, B:83%, None:0%) and 2-AG+PEA (N:29%, C:2%, B:66%, None:2%) after 24 h. PEA (N_1 *h*_:51%, C_1 *h*_:5%, B_1 *h*_:17%, None_1 *h*_:27%; N_6 *h*_:61%, C_6 *h*_:7%, B_6 *h*_:18%, None_6 *h*_:14%), 2-AG (N_1 *h*_:67%, C_1 *h*_:3%, B_1 *h*_:29%, None_1 *h*_:1%; N_6 *h*_:30%, C_6 *h*_:11%, B_6 *h*_:34%, None_6 *h*_:26%) and PEA+2-AG (N_1 *h*_:54%, C_1 *h*_:14%, B_1 *h*_:20%, None_1 *h*_:12%; N_6 *h*_:21%, C_6 *h*_:21%, B_6 *h*_:35%, NS_6 *h*_:23%) changed significantly the localization of PPARα after 1 and 6 h in combination with LPS (vs. LPS; N_1 *h*_:25%, C_1 *h*_:15%, B_1 *h*_:53%, None_1 *h*_:7%; N_6 *h*_:67%, C_6 *h*_:10%, B_6 *h*_:20%, None_6 *h*_:3%). No effect could be observed for LPS+PEA (N: 22%, C: 3%, B: 69%, None: 6%) in comparison to LPS (N: 28%, C: 2%, B: 56%, None: 14%) after 24 h, but 2-AG (N: 27%, C: 2%, B: 69%, None: 2%) and 2-AG+PEA (N: 20%, C: 0%, B: 80%, None: 0%) if co-applied with LPS altered significantly the distribution (Figures 4A–C and Table 2).

**TABLE 2:** PPARα subcellular distribution and cellular localization after incubation with 2-AG and PEA in primary microglial cells.

	**Mean**	**SEM**	**Mean**	**SEM**	**Mean**	**SEM**	**Mean**	**SEM**	**Mean**	**SEM**	**Mean**	**SEM**	**Mean**	**SEM**	**Mean**	**SEM**
								
**Localization**	**CTL**		**PEA**		**2-AG**		**2-AG+PEA**		**LPS**		**LPS+PEA**		**LPS+2-AG**		**LPS+2-AG+PEA**	
**1 h**																
Nucleus	0.53	0.01	0.39	0.01	0.30	0.01	0.34	0.01	0.18	0.01	0.26	0.02	0.65	0.01	0.38	0.01
Cytoplasm	0.04	0.00	0.06	0.00	0.18	0.01	0.03	0.01	0.10	0.00	0.03	0.01	0.03	0.00	0.10	0.00
Both	0.24	0.01	0.26	0.01	0.28	0.01	0.29	0.01	0.36	0.01	0.09	0.01	0.29	0.01	0.14	0.01
No signal	0.20	0.01	0.03	0.00	0.09	0.00	0.03	0.01	0.05	0.00	0.14	0.01	0.01	0.00	0.09	0.00
**6 h**																
Nucleus	0.35	0.01	0.51	0.01	0.22	0.01	0.20	0.01	0.51	0.01	0.53	0.01	0.27	0.01	0.18	0.01
Cytoplasm	0.02	0.00	0.12	0.01	0.16	0.01	0.10	0.01	0.08	0.01	0.06	0.01	0.10	0.01	0.18	0.01
Both	0.63	0.01	0.39	0.01	0.37	0.01	0.45	0.01	0.16	0.01	0.16	0.01	0.31	0.01	0.29	0.01
No signal	0.00	0.00	0.02	0.00	0.08	0.01	0.14	0.01	0.02	0.00	0.12	0.01	0.24	0.01	0.20	0.01
**24 h**																
Nucleus	0.23	0.01	0.50	0.01	0.13	0.01	0.30	0.01	0.30	0.01	0.18	0.01	0.35	0.01	0.23	0.01
Cytoplasm	0.03	0.00	0.03	0.00	0.08	0.01	0.03	0.00	0.03	0.00	0.03	0.01	0.03	0.00	0.00	0.00
Both	0.75	0.01	0.35	0.01	0.95	0.01	0.68	0.01	0.60	0.01	0.55	0.01	0.90	0.01	0.90	0.01
No signal	0.00	0.00	0.10	0.01	0.00	0.00	0.03	0.00	0.15	0.01	0.05	0.01	0.03	0.00	0.00	0.00

*Rounded mean values (two positions) and SEM.*

Lipopolysaccharide alone induced a shift to nuclear localization after 6 h, whereas LPS+PEA+2-AG after 6 h led to more cytoplasmic expression, similar to 2-AG and PEA alone (Figures 4D,E).

## Discussion

Reduction of neuronal damage in injured patients is a main contributor to sustain quality of life, for this reason its improvement and new approaches are needed. Evidence has accumulated that endocannabinoids can be beneficial for treatment of patients with brain injury (Fernandez-Ruiz et al., 2010). Cannabinoids, as shown before have different targets and are potential therapeutics, but first it is necessary to better understand the molecular and cellular mechanisms of cannabinoid action.

### The Protective Effects of 2-AG and PEA Are Abolished, When Both Are Applied Together

In this study, we focused on excitotoxicity, a main factor of neuronal damage as described before (Kunz et al., 2010; Lau and Tymianski, 2010). To investigate the intrinsic responses and to exclude interfering influences such as blood flow and infiltrating peripheral immune cells the well-established model of excitotoxic lesioned OHSC was chosen. Both, PEA and 2-AG were shown to be protective with microglia participation in NMDA lesioned OHSC before. In our previous studies we extensively examined the involvement of cannabinoid receptors in PEA and 2-AG mediated actions. Whereas PEA and the synthetic PPARα agonist Wy-14,643 protected dentate gyrus granule cells, treatment with the PPARα antagonist GW6471 blocked PEA-mediated neuroprotection. Selective activation or inhibition of PPARγ displayed no positive effect (Koch et al., 2011a). Interestingly, 2-AG induced neuroprotection was inhibited by cannabidiol (CBD) and O-1918 and mimicked by abn-CBD indicating the involvement of abn-CBDR. The 2-AG effects were not blocked by the specific CB_2_ receptor antagonist (AM630). Depletion of microglial cells abolished the neuroprotection mediated by 2-AG or abn-CBD raising the hypothesis that the neuroprotective effects of 2-AG were abn-CBDR and microglia dependent (Kreutz et al., 2007, 2009). The abn-CBDR is a pharmacologically characterized non-CB_1_/non-CB_2_ receptor and has been first described on endothelial cells of rat mesenteric blood vessels (Járai et al., 1999; Pertwee et al., 2010), and has not been identified yet. PEA, 2-AG or their analogs were also protective in other models like moderate traumatic injury and reduced neuroinflammation (Panikashvili et al., 2001; Genovese et al., 2008; D’Agostino et al., 2012; Esposito et al., 2012; Mounsey et al., 2015; Guida et al., 2017; Belardo et al., 2019; Impellizzeri et al., 2019). In PPARα^–/–^ mice PEA induced neuroprotection after spinal cord trauma was abolished, however involvement of further PPARs was suggested (Paterniti et al., 2013). The application of PEA was found to trigger the synthesis of 2-AG in human and canine plasma and enhance its effects on transient receptor potential cation channel subfamily V member 1 (TRPV1) in HEK-293 cells (Petrosino et al., 2016). In striatum PEA triggered the synthesis of 2-AG via GPR55 (Musella et al., 2017). These effects were referred to an entourage effect, which might be mediated through TRPV1 activation or inhibition of fatty acid amide hydrolase, with PEA as its substrate or activation of GPR55 (Jonsson et al., 2001; Smart et al., 2002). A further possible explanation is an allosteric modulation, which could explain that 2-AG and PEA abolished their neuroprotective properties in the OHSC model and reduced ramification index at PEA level. While TRPV1 was not found in OHSC, GPR55 was expressed in slices and was associated with neuroprotection (Grabiec et al., 2012; Kallendrusch et al., 2013). Consequently, GPR55 might be involved in the PEA mediated actions observed here. Furthermore, PEA was also shown to enhance the CB_2_ expression via PPARα activation in macrophages (Guida et al., 2017). Increased number of CB_2_ next to abn-CBDR can be activated by 2-AG and induce further effects. PEA at cellular level reduced ramification index, making cells more ramified whereas 2-AG had no impact on morphology of microglial cells. The decrease in neuroprotection mediated by co-treatment with both cannabinoids might be attributable to their effects on cellular level, especially on microglial cells, the conductor of the neuroprotective properties of 2-AG and PEA. An entourage effect with PEA as enhancer of the anti-inflammatory and anti-nociceptive activity of other endogenous compounds by potentiating their affinity for a receptor or by inhibiting their metabolic degradation (Skaper and Facci, 2014) is missing in OHSC in the context of neuroprotection.

### Effects of PEA and 2-AG Are Microglia Dependent

Neuroprotective capabilities of microglial cells include synaptic stripping, induction of neurogenesis, phagocytosis, and maintenance of central nervous system homeostasis (Chen and Trapp, 2016). On the other hand microglial cells are also able to suppress neuroinflammation, protect nerve tissue by producing anti-inflammatory and tissue-repairing cytokines and factors (Colton, 2009). Microglial cells can contribute to neuronal damage via inflammation and release of cytotoxic substances as described earlier and are associated with neurodegenerative diseases, cognitive dysfunction in aging and dementia, epilepsy, and other conditions leading to brain inflammation and neuronal lesion (Ekdahl et al., 2003; Smith et al., 2012; Chhor et al., 2013). Cannabinoids were shown to interact with microglial cells in further pathological states and to change the morphology, activation and number of the cells (Zhang et al., 2003; Luongo et al., 2010; Guida et al., 2017).

After microglia depletion in OHSC with the bisphosphonate clodronate PEA and 2-AG lost their neuroprotective effects supporting the hypothesis that 2-AG, PEA exert their neuroprotective effects via microglial cells. PEA was shown before to induce migration and increase of motility of BV2 microglial cell line (Franklin et al., 2003; Guida et al., 2017). Notably, microglial cells produce both 2-AG and PEA (Muccioli et al., 2007; Muccioli and Stella, 2009) and their levels were found to be increased after focal cerebral ischemia (Franklin et al., 2003).

### PEA and 2-AG Mediate Opposite Effects on Nitric Oxide Synthesis in Primary Microglial Cells

Endocannabinoids, which are in part produced by microglia, affect the ability of microglia cells to proliferate, phagocytize, and produce NO (Stella, 2009). NO production occurs enzymatically via a conversion of L-arginine to L-citrulline by NO synthase, competing with arginase for L-arginine as a substrate. iNOS is not expressed in healthy brain tissue under physiological conditions but its expression can be induced in astrocytes and microglial cells through trauma. iNOS induction starts several hours before NO is generated and involves transcription of mRNA and novel protein synthesis. iNOS is mainly expressed under inflammatory conditions and after transient ischemic periods and is known as an antimicrobial defense mechanism of the immune system (Vincent et al., 1998). The increase in NO driven by iNOS might be neurotoxic as it forms reactive nitrogen oxide species like peroxynitrite (Garry et al., 2015). It was reported that 2-AG alone stimulates the release of NO from human immune and vascular tissues and from invertebrate immune cells via CB_1_ activation (Stefano et al., 2000). Contrary, in our study 2-AG or PEA applied alone had no effect on NO production by microglial cells after 72 h. However, LPS in combination with 2-AG increased NO concentration, although 2-AG was protective after NMDA damage. PEA was able to reduce NO production by microglial cells after LPS treatment. In accordance to this study PEA significantly inhibited the NO release induced by LPS in murine macrophage cell line RAW264.7, which was not sensitive to pertussis toxin treatment, indicating a G-protein independent mode of action (Ross et al., 2000). Pre-treatment of LPS-stimulated primary mouse microglial cells with PPARα agonists also resulted in inhibition of NO production (Xu et al., 2005). In similarity to PEA in murine macrophage cell line RAW264.7, WIN55,212, a neuroprotective cannabinoid decreased NO level when applied together with LPS via CB_2_ receptor (Ross et al., 2000; Koch et al., 2011b). Furthermore, PEA reduced iNOS expression in spinal cord 6 h after paw edema induction (D’Agostino et al., 2007). The results presented in our study are in accordance with previous investigations and strengthen the hypothesis that PEA mediates neuroprotection via microglial cells. The neuroprotective effects observed here in the OHSC model underline the intrinsic positive effects of cannabinoids as influences through alterations in cerebral blood flow and infiltration of peripheral cells are missing (Grabiec et al., 2017), making the inhibition of NO produced by microglial cells one possible explanation for neuroprotection. In agreement with our work and previous studies LPS induces NO production mainly in microglial cells in models of excitotoxicity or during transient ischemic periods (Nakamura et al., 1999; Genovese et al., 2008; Yao et al., 2017). Further endocannabinoids were shown to decrease iNOS activity in rat microglial cells, e.g., CP55940 exerted a dose-dependent inhibition of interferon gamma/LPS-inducible NO production (Cabral et al., 2001) Little is known about the influence of endocannabinoids on arginase, in peripheral immune cells and in microglial N9 cells Δ-9- tetrahydrocannabinol and CB_2_ agonist induced arginase 1 expression, what reduce NO production (Hegde et al., 2010; Ma et al., 2015).

Through combination treatment of both PEA and 2-AG, NO production was significantly elevated and neuroprotective properties were diminished. It leads to the hypothesis that PEA may be neuroprotective through reduction of NO present after brain injury. On the other hand, 2-AG significantly elevated NO production while still exerting neuroprotective effects similar to NO donors (Kakizawa et al., 2007), 2-AG acts probably via another targets and mechanisms.

### Changes in Morphology and Phenotypes of Primary Microglial Cells After Treatment

*In vivo*, *ex vivo*, and *in vitro* a broad spectrum of differentiation stages of microglial cells has been observed (Dubbelaar et al., 2018). Under physiological conditions microglial cells are ramified and constantly scanning their surroundings (Nimmerjahn et al., 2005). Upon damage or inflammation, they become amoeboid, move to the site of neuronal lesion and remove cell debris. However, this morphological change of microglia upon a shift in activation state does not seem to be uniform, but it can be best mimicked by administration of LPS. The morphology ranges from amoeboid-like shapes during inflammation to highly ramified ones and includes many intermediate forms that are often associated with the activation status of microglial cells (Zhou et al., 2017; Dubbelaar et al., 2018). According to the phenotype microglial cells were often described as anti-inflammatory or neuroprotective or proinflammatory and neurotoxic. However, there is no binary system of phenotypes for microglia and they may possess multiple context dependent properties at the same time (Ransohoff, 2016; Dubbelaar et al., 2018).

In synopsis with NO reduction, the administration of PEA to LPS stimulated microglial cells induced a more ramified morphology; these anti-inflammatory properties of PEA may mediate its neuroprotective effects. 2-AG did not alter the LPS induced amoeboid state of microglial cells but increased the NO concentration, indicating that the neuroprotective properties of 2-AG are mediated through other microglial cell dependent mechanisms than those involving NO. However, *in vivo* and in acute experimental autoimmune encephalomyelitis 2-AG increased the ramification of microglia (Lourbopoulos et al., 2011). In excitotoxically lesioned OHSC 2-AG reduced the number of microglial cells (Kreutz et al., 2007). It is plausible that the absence of changes is model dependent or that 2-AG influenced other properties of microglia, which were not examined in this study, like phagocytosis, motility, and alterations in extracellular signaling.

The combination of 2-AG and PEA induced a ramification of microglial cells in comparison to 2-AG and abolished PEA mediated NO concentration decrease. This may indicate that the anti-inflammatory properties of PEA are lost if co-incubated with 2-AG, since decrease in the number of damaged neurons in OHSC was missing and therefore neuroprotection. 2-AG and PEA exert contrary effects on microglial cells and it is plausible, that co-application of both led to their abolishment. Expected entourage effect is missing in case of this model and those substances.

It will be necessary to verify how 2-AG exerts neuroprotection at a cellular level and whether this process is microglia dependent only, since 2-AG was found protective in isolated neuronal cultures (Zhang and Chen, 2008). Interestingly, proliferation of pure microglial cell cultures was not affected in this study, whereas the number of microglial cells was significantly decreased in dentate gyrus in OHSC after excitotoxical damage and treatment with PEA or 2-AG, this effect was mimicked by PPARα agonists and blocked by its antagonists (Kreutz et al., 2007; Koch et al., 2011a). Since microglial cells in culture proliferate very slowly without astrocytes, it seems plausible that the effects observed in OHSC might be the result of PEA actions on astrocytes, microglia, possibly neurons in the network and/or their cross-talk, since all cell types express the PPARα receptor (Xu et al., 2006; Warden et al., 2016).

### PPARα Expression and Localization Changed After Incubation With Cannabinoids

Peroxisome proliferator-activated receptors predominantly are localized in the nucleus, theirs activity is modulated via phosphorylation and PPARα undergoes ligand-dependent nucleo-cytoplasmic shuttling (Burns and Vanden Heuvel, 2007; Umemoto and Fujiki, 2012). The amount of PPARα was shown to increase in microglial cells in nucleus after 6 h and to decrease after 24 h after PEA treatment. PPARs are ligand-activated transcription factors, and their biological role is coupled to the function of their target genes. In immunocytochemical staining a translocation of PPARα into the nucleus was seen. Neuroprotective effects of PEA are known to be mediated by PPARα. On the one hand, the nuclear localization of the receptor induces specific PEA related effects; its shift into the cytoplasm reduces the amount of available receptor in the nucleus. On the other hand the cytoplasmic localization of PPARα may favor the binding of other ligands and mediate different actions (Guida et al., 2017). The lower levels of PPARα in nuclei of microglial cells after simultaneous treatment with 2-AG and PEA with or without LPS might be a possible explanation for the missing neuroprotection in OHSC. It also indicates an interference between 2-AG and PEA signaling pathways with a more dominant role of 2-AG and inhibitory actions on PPARs translocation into the nucleus. Specific activation of central PPARα controls inflammation in the spinal cord as well as in the periphery (D’Agostino et al., 2007), when the amount of receptor decreased no effect could be induced. Additionally, fatty acid oxygenase metabolism products of 2-AG were able to activate PPARα receptor in human undifferentiated epidermal keratinocytes and stimulation of PPARα and its downstream target genes led to cell differentiation (Kömüves et al., 2000; Kozak et al., 2002). It is plausible that 2-AG and PEA interact directly on PPARα receptor in the opposite way.

## Conclusion

While endocannabinoids are promising regarding treatment of neuronal diseases, and their neuroprotective properties are known for a while, little is known how they are mediated. 2-AG and PEA are both protective agents with different targets. Their positive effect is not enhanced by co-incubation. The understanding of interactions between signaling pathways of different endocannabinoids will help to elucide the in part conflicting results reported in the literature.

## Data Availability Statement

All datasets generated for this study are included in the article/supplementary material.

## Ethics Statement

The animal study was reviewed and approved by the local authorities of the State of Saxony-Anhalt (permission number: I11M18, date: 01.12.2012) protecting animals and regulating tissue collection used for scientific purposes.

## Author Contributions

FD and UH: conceptualization, supervision, and project administration. MP, UH, TH, and JK: methodology and formal analysis. TH: software and data curation. MP, UH, TH, and FD: validation. MP, UH, JK, TH, and CG: investigation. FD: resources. UH and MP: visualization and writing original draft. UH, TH, JK, and FD: writing review and editing. All authors contributed to the manuscript revision, and read and approved the submitted version.

## Conflict of Interest

The authors declare that the research was conducted in the absence of any commercial or financial relationships that could be construed as a potential conflict of interest.

## Abbreviations

2-AG: 2-arachidonoylglycerol
abn-CBD: abnormal cannabidiol
abn-CBDR: abn-CBD-sensitive receptor
CB: cannabinoid receptor
CBD: cannabidiol
div: day *in vitro*
iNOS: inducible nitric oxide synthase
LPS: lipopolysaccharide
NMDA: *N*-methyl -D -aspartate
NO: nitric oxide
OHSC: organotypic hippocampal slice cultures
PEA: palmitoylethanolamide
PPAR: peroxisome proliferator-activated receptor.
